# Using virtual reality to train emotional and social skills in children with autism spectrum disorder

**DOI:** 10.1080/17571472.2018.1483000

**Published:** 2018-06-07

**Authors:** Sze Ngar Vanessa Yuan, Horace Ho Shing Ip

**Affiliations:** a Centre for Innovative Applications of Internet and Multimedia Technologies AIMTech Centre, City University of Hong Kong, Kowloon, Hong Kong

**Keywords:** Autism spectrum disorder, social skills, emotions, virtual reality, child

## Abstract

With emerging technology, computerised, internet-based and virtual reality (VR)-based treatment and training became increasingly popular. VR provides an immersive experience into a simulated environment. Autism spectrum disorder (ASD) is characterised by social communication deficits and repetitive behaviours. Children with ASD often require social skills training while VR provides a safe, controllable environment to practice skills repeatedly. The Centre for Innovative Applications of Internet and Multimedia Technologies (AIMTech Centre) at City University of Hong Kong developed a VR-enabled training program to examine its efficacy on emotional and social skills with six VR scenarios depicting the daily lives of typical children in Hong Kong. 94 children from mainstream primary schools in Hong Kong completed the study and 72 children were included in the analysis. Children from training group scored higher on emotion expression and regulation (*M* = 20.2, SD = 3.00) than before the training (*M* = 18.9, SD = 3.57, *t*(35) = −2.174, *p* = .037) and higher on social interaction and adaptation after the training (*M* = 21.8, SD = 2.99) than before training (*M* = 20.2, SD = 3.43, *t*(35) = −3.987, *p* < .0005). There was a statistically significant interaction between group and time on affective expressions, *F*(1, 70) = 5.223, *p* = .025, partial *η*^2^ = .069, and on social reciprocity, *F*(1, 70) = 7.769, *p* = .007, partial *η*^2^ = .100. Children were able to engage in VR training despite initial challenges with viewing goggles. Some children declined to participate due to scheduling reasons which may be minimised through the adoption of head-mounted displays as a portable, cost-effective device. VR seems to be a promising asset to traditional training and therapy while the importance trainers’ or therapists’ support has yet to be further investigated.

## Why this matters to me

When I first moved back to Hong Kong from the United Kingdom, I was clueless about the culture in Hong Kong. After completing a postgraduate diploma in education, focusing on special education, and having worked at AIMTech Centre on a research project on autism spectrum disorder (ASD) for three years, I became increasingly aware of the difficulties and challenges faced by children with ASD, as well as teachers and professionals working with this population. I have also learnt a lot about virtual reality (VR) and have designed a whole training program from original ideas into a comprehensive training applying the technology of VR into traditional approaches. I really believe that VR has great potential in education and in the mental health field.

## Key message

Virtual reality supports training and therapy for children with autism spectrum disorder.

## Governance

City University of Hong Kong.

## Evidence of permissions to publish other people’s work

Permission from my work supervisor, Professor Horace H. S. Ip, at AIMTech Centre.

## Introduction

Emerging new technology contributes to our understanding and offers new solutions to current phenomena. For instance, the increased popularity of computerised and internet-based treatment for depression was found to be moderately effective given therapist support is included with the computerised treatment [1]. In more recent years, an increasing number of studies on virtual reality (VR)-based training and therapy have established good evidence on its facilitation of traditional approaches. VR simulates different senses for an immersive experience. Its application on mental health disorders and developmental disorders has been increasingly researched. The development of a virtual reality environment (VRE) offers a promising training tool in an immersive, computer-generated virtual world. VRE can be created to simulate real-life situations while it is also possible to create an environment that is rarely or never found in real life. For instance, a snow environment would be something that a child in Hong Kong has unlikely experienced in real life before, while a dancing snowman could be the first experience to anyone immersing into the VRE.

Autism spectrum disorder (ASD) is a developmental disorder characterised by persistent impairment in social communication and patterns of repetitive behaviours [2]. Social stories are useful in teaching children with ASD about social situations and formulating socially appropriate responses [3]. It minimises potentially confusing instructions by providing direct social information, followed by new skills practices. Traditionally, social stories are presented on paper, as a recording or as a video, followed by role-play or subsequent practices. However, not all social situations could be well-practiced in real life. For example, it is not safe and humane to have a pedestrian crossing the red light in real life to teach children the right behaviour and appropriate responses. However, such situation could be programmed into a VRE, providing a safe, controllable environment to practice skills repeatedly. A recent meta-analysis revealed that innovative technology interventions have a moderate effect (*d* = .047) in the post-tests of the targeted skills in children with ASD, supporting the development, research and clinical use of such intervention [4].

## Method

Based on the challenges experienced by children with ASD and their teachers, the Centre for Innovative Applications of Internet and Multimedia Technologies (AIMTech Centre) at City University of Hong Kong has developed a VR training program to help children with ASD. The immersive VRE was delivered in a four-side Cave Automatic Virtual Environment (CAVE) allowing for great fidelity and interaction with objects and avatars in virtual scenarios covering real-life situations. During the training, a group of 3 to 4 children would gather for a briefing session to review previously learnt skills and concepts, and prepare for the VR training. Then, the children would enter the CAVE individually and navigate in the VRE with guidance and support from the trainer. The one-hour training session would end with a debriefing session to discuss their experience and generalise their learnt skills into real life. Six learning scenarios were designed, including a relaxation scenario, four training scenarios and one consolidation scenario. The scenarios were designed to best match the real life of a typical primary school-aged child in Hong Kong.

Primary school-aged children with diagnoses of ASD were eligible to participate in the study. A total of 127 children were referred by 16 local mainstream primary schools across Hong Kong. 3 children were lost to contact, 1 child declined to participate and 29 were unable to participate due to scheduling reasons. Parents and children were invited to an information session while the parents provided written consent for their child to participate in the study. All personal information was kept confidential. The children and parents understood that their participation was voluntary and that they reserved the rights to withdraw from the study at any time.

Psychoeducational Profile, Third Edition (PEP-3) assesses skills and behaviours in children with ASD [5]. The subtests of affective expressions and social reciprocity were used to investigate the training’s effect on the primary outcomes of emotion expression and regulation, and social interaction respectively [5]. Other than quantitative data, on-going qualitative data from communication log and in-class observation log are retrieved.

## Results

94 children completed the study and 72 children were included in the analysis after filtering for age. There were 64 boys (88.9%) and 8 girls (11.1%) with a mean age of 106.3 months (SD = 13.53; Table 1). Children with documentation of intellectual disability were excluded from this study. The children have all received a diagnosis of ASD by Child Assessment Service in Hong Kong or via private practice. A waitlist control design was adopted.

**Table 1.** Descriptive statistics of included participants.

	Training (*n* = 36)	Control (*n* = 36)	Training vs. Control
	*n* (%)	*n* (%)	*p*
Sex (male)	31 (86.1)	33 (91.7)	.453
	*M* (SD)	*M* (SD)	
Age (months)	107.6 (13.27)	104.8 (13.83)	.769

Paired sample t-test results have shown that children from training group scored higher on emotion expression and regulation after the training (*M* = 20.2, SD = 3.00) than before the training (*M* = 18.9, SD = 3.57, *t*(35) = −2.174, *p* = .037) [6]. Children from training group also scored higher on social interaction and adaptation after the training (*M* = 21.8, SD = 2.99) than before training (*M* = 20.2, SD = 3.43, *t*(35) = −3.987, *p* < .0005) [6]. Mixed repeated measures ANOVA results revealed that there was a statistically significant interaction between group and time on affective expressions, *F*(1, 70) = 5.223, *p* = .025, partial *η*^2^ = .069 [6]. There was also a significant interaction between group and time on social reciprocity, *F*(1, 70) = 7.769, *p* = .007, partial *η*^2^ = .100 [6]. Tables 2 and 3 show a summary of the results respectively.

**Table 2.** Comparison of pre- and post-training scores within training group.

Measures	Training (*n* = 36)				
	Pre-training	Post-training	*d*	*t*	*p*
	*M* (SD)	*M* (SD)			
PEP-3 affective expressions	18.9 (3.57)	20.2 (3.00)	.394	−2.174	.037
PEP-3 social reciprocity	20.2 (3.43)	21.8 (2.99)	.497	−3.987	<.0005

**Table 3.** Mixed repeated measures ANOVA results.

Measures	df (between)	df (within)	*F*	*p*	Partial *η*^2^
PEP-3 affective expressions	1	70	5.223	.025	.069
PEP-3 social reciprocity	1	70	7.769	.007	.100

Qualitative feedbacks were received from an ongoing communication log with parents and teachers. Many parents expressed that their children were much more proactive in greeting and communicating with neighbours and relatives. Some parents reported that their children became more flexible in either their seat preferences or food preferences. Teachers also reported seeing students beginning to make more new friends and engaging in two-way conversations.

Children were all able to engage in the VR training despite a few initial reluctances in wearing the stereotypic viewing goggles. The children refused to wear the goggles due to previous bad experience (e.g. at the 3D movies) or uncertainties. After the trainer’s reassurance and support, all children were willing to wear the goggles after no more than three sessions. The children understood both visual and audio information in the VR system, but the trainer plays a huge role in facilitating the training, particularly in dealing with any emotional or behavioural issues that may arise during the training. The briefing and debriefing sessions were essential as a gateway to bridge and generalise learnt skills from VR to reality.

The biggest reason for declining to participate was due to scheduling reasons. CAVE is a huge and expensive VR-enabling technology. It requires an enormous amount of space and a high development and maintenance cost. A typical school or organisation could not afford to build one. The participants of this study must travel to the CAVE at City University of Hong Kong in this study, which may be a burden given the packed schedule of a typical primary student and a working parent.

## Discussion

With such promising results, a subsequent project has begun in late 2017 with the use of head-mounted display (HMD) to address the inconvenience and cost of this presented study. HMDs are relatively mobile and could be installed in any location with power socket. The training would be delivered in schools to benefit a much larger population. Other than social and emotional skills, VR can also be applied to other disorders, such as anxiety, or as a medium to raise public concerns such as reducing stigma. It is important to consider ways to increase the convenience of VR application to popularise it in more communities. VR through mobile phone application along with VR cardboards may be plausible, but one must consider the importance of therapists’ or trainers’ support in therapies and psychoeducational training.

## Disclosure statement

No potential conflict of interest was reported by the authors.

## Funding

This work was supported by Quality Education Fund, Hong Kong [Project number 2013/0223].
